# Logarithmic Helical Design for Reversed Magnetic Field in Magnetoelastic Soft Matters with Giant Current Outputs

**DOI:** 10.1002/advs.202505157

**Published:** 2025-05-11

**Authors:** Xiaojun Chen, Farid Manshaii, Dianyu Tang, Yizhuo Xu, Zhuofan Li, Manhui Chen, Peng Chen, Yike Li, Shanfei Zhang, Lei Yang, Jun Chen, Bin Su

**Affiliations:** ^1^ State Key Laboratory of Materials Processing and Die & Mould Technology School of Materials Science and Engineering Huazhong University of Science and Technology Wuhan 430074 People's Republic of China; ^2^ Department of Bioengineering University of California Los Angeles CA 90095 USA; ^3^ School of Transportation and Logistics Engineering Wuhan University of Technology Wuhan Hubei 430063 People's Republic of China; ^4^ State Key Laboratory of Advanced Electromagnetic Engineering and Technology School of Electrical and Electronic Engineering Huazhong University of Science and Technology Wuhan Hubei 430074 People's Republic of China

**Keywords:** energy harvesting, helical structure, magnetic field variation, magnetoelasticity, wearable knee pad

## Abstract

Magnetoelastic soft materials are widely used in soft bioelectronics. However, mechanical deformation usually induces minimal changes in magnetic flux, limiting electrical outputs. To overcome this limitation, a two‐step process is employed to enhance the variation in magnetic flux density under mechanical force. On one hand, the helical structural design enables the magnetic membrane to flip completely, reversing the magnetic field. On the other hand, the applied mechanical force induces strain within the magnetoelastic membrane, leading to variations in magnetic flux density. A complete 180° reversal of the magnetic field is achieved using a logarithmic helical structure, resulting in a 200% increase in magnetic flux variation and a peak current of 6.34 mA. Following structural optimization, the current density reached an impressive 7.17 mA cm^−2^. Using this rationally designed logarithmic helix model, a knee pad is developed for wearable energy harvesting from human body movement. The device can generate a current of up to 2.83 mA, providing sufficient power for various small electronics, including smartphones, LED lights, headlamps, and rechargeable batteries. This achievement represents a significant milestone in advancing high‐performance wearable biomechanical energy harvesting.

## Graphical abstract

According to literatures, magnetoelastic soft materials have achieved a maximum magnetic flux change of 80%, making it difficult to enhance the magnetic flux change. To address this, this manuscript proposed a reversed magnetic field strategy in conjunction with logarithmic helical design and magnetoelastic deformation. Consequently, reversed magnetic field sample was fabricated, achieving a 200% change in magnetic flux.

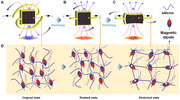

## 1 Introduction

Magnetoelastic soft materials, composed of magnetic nanoparticles embedded in soft matter systems, have attracted significant attention since its invention,^[^ ^1^ ^]^ owing to their collection of compelling advantages, including being intrinsically waterproof,^[^ ^2^ ^]^ biocompatible,^[^ ^3^ ^]^ stretchable,^[^ ^4^ ^]^ cost‐effective, and with self‐powered working mode.^[^ ^5^ ^]^ These magnetoelastic materials show promise in various applications, such as flexible electronics,^[^ ^6^ ^]^ electronic skin,^[^ ^7^ ^]^ textile bioelectronics,^[^ ^8^ ^]^ even implantable bioelectronics,^[^ ^9^ ^]^ for biomonitoring,^[^ ^10^ ^]^ therapeutics,^[^ ^11^ ^]^ and energy.^[^ ^12^ ^]^ The pronounced magnetoelastic effect observed in soft systems arises from microstructure changes of the micromagnetic chains under mechanical deformation. Specifically, when external stress is applied to a magnetoelastic material, its internal magnetic wavy chains reconfigure to accommodate the new stress conditions, leading to a variation in magnetic flux density. However, this pressure‐induced material deformation and the resulting magnetic field variation are very limited, largely limiting the overall electrical output. Achieving a more significant variation in magnetic flux under mechanical force remains highly desired.

In this work, we report a two‐step procedure to increase the magnetic field variation under mechanical force by using a magnetoelastic composite with a logarithmic helical structure. The applied mechanical force initially flips the helical magnetoelastic soft materials, generating an inverted magnetic field and achieving a complete 180° magnetic field reversal. Then, the mechanical force will further cause strain change within the magnetoelastic soft materials to induce a secondary magnetic field variation. Both simulations and experiments confirmed that the logarithmic helical structure could achieve a 200% increase in the magnetic flux variation, resulting in an electrical output of up to 6.34 mA and a current density of 7.17 mA/cm^2^. With this unique two‐step mechanical to magnetic conversion, a wearable knee pad was developed to harvest energy from human body motion. It has been shown to efficiently power a variety of electronic devices, such as smartphones, LED lights, headlamps, and rechargeable batteries. The logarithmic helical structural design, coupled with a two‐step conversion process, offers a promising solution for wearable electricity generation.

## 2 Results and Discussion

### 2.1 Design Strategy for Logarithmic Helical Structures

Current design systems for magnetoelastic soft materials primarily induce changes in magnetic flux through structural deformation, thereby altering the surrounding magnetic field.^[^ ^13^ ^]^ The change in magnetic flux is primarily due to the deformation‐induced shift in the relative positions of the magnetic nanoparticles within the magnetoelastic material. This shift alters the orientation and distance between magnetic nanoparticles, leading to a variation in the magnetic flux. However, such deformations usually occur at a localized level, making it difficult to achieve a broad variation in the magnetic field. As a result, significantly enhancing magnetic flux changes and improving energy conversion efficiency becomes challenging. Achieving a large‐angle rotation of the magnetic field over a wide area could greatly enhance both power generation and signal strength. Literature indicates that, in certain representative studies,^[^ ^1^, ^10^, ^12^, ^14^ ^]^ magnetoelastic soft materials have achieved a maximum flux change of 80%^[^ ^12b^ ^]^ (Table S1, Supporting Information), making it challenging to reach 100% flux change.

**Figure 1:**
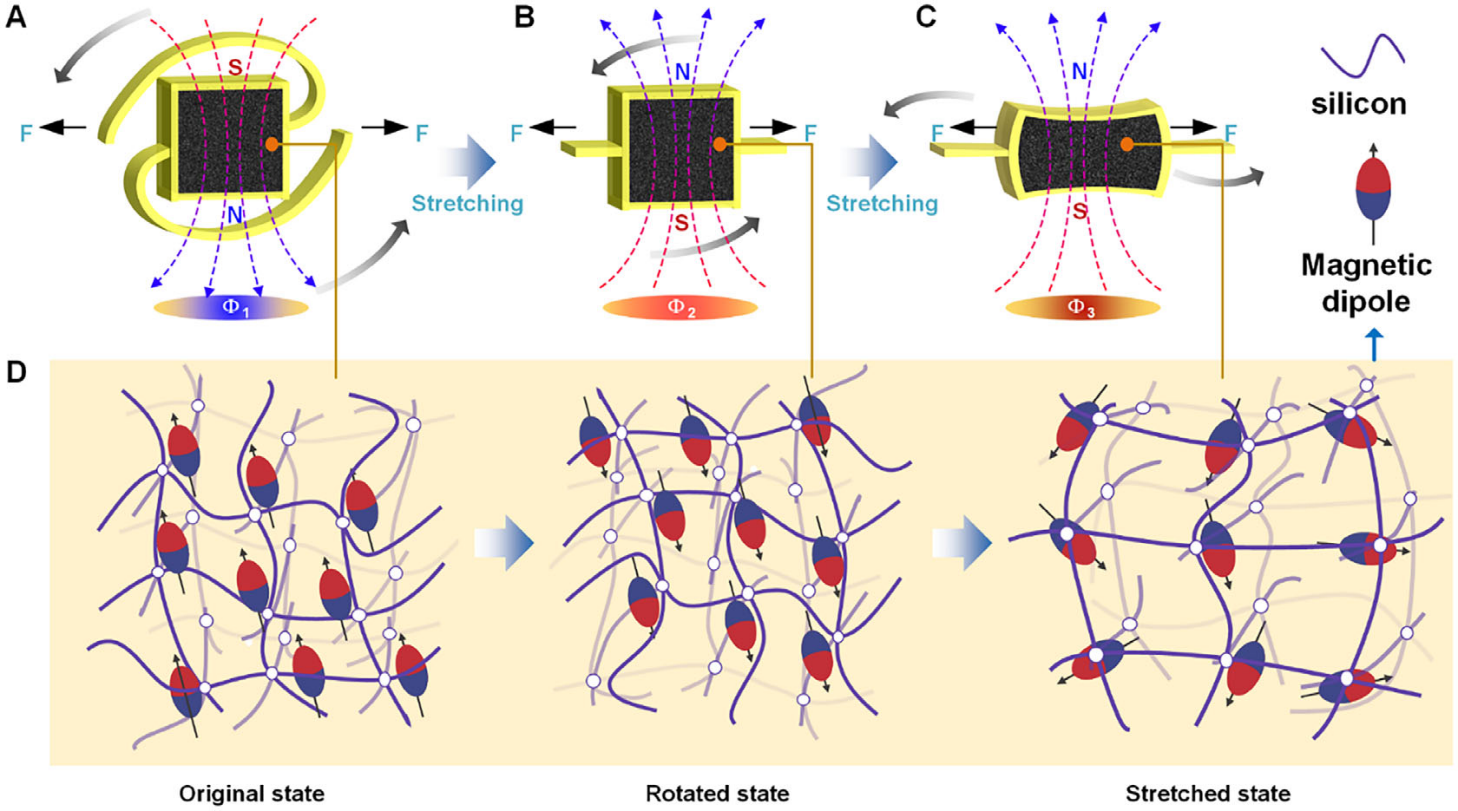
Logarithmic helical structure design combined with magnetoelastic deformation in a two‐step procedure for enhanced magnetic field variation and energy conversion. A) Unstretched state of the designed logarithmic helical structure. B) Logarithmic helical structure stretched to a state where the center magnetic block is rotated 180°. C) The logarithmic helical structure continues to be stretched to a state where the magnetoelastic block in the middle undergoes deformation. D) Schematics of the magnetic dipole distribution in magnetoelastic block (corresponding to Figures A, B, and C).

Based on this, the characteristics of the magnetoelastic soft material are combined with the logarithmic helical structure rotation strategy to regulate changes in magnetic flux by rotating the magnetic field over a large angle. This regulation enables a change in the direction of the magnetic flux. As shown in **Figure** **1**, the combination of the logarithmic helical structure design and magnetoelastic deformation facilitates efficient energy conversion through a two‐step strategy. Specifically, first, under stretching conditions, the overall 180° rotating effect of the magnetic field is achieved through the helical structure design (Figure 1A,B). Second, continued stretching induces magnetoelastic deformation of the magnetic material (Figure 1C). The schematic representation of changes in magnetic field variation within the magnetoelastic material during the two‐step process is illustrated in Figure 1D. Ultimately, the strategy proposed in this work can achieve a 200% change in magnetic flux, further leading to efficient energy conversion.

### 2.2 Fabrication of Reversed Magnetic Field Samples

**Figure 2:**
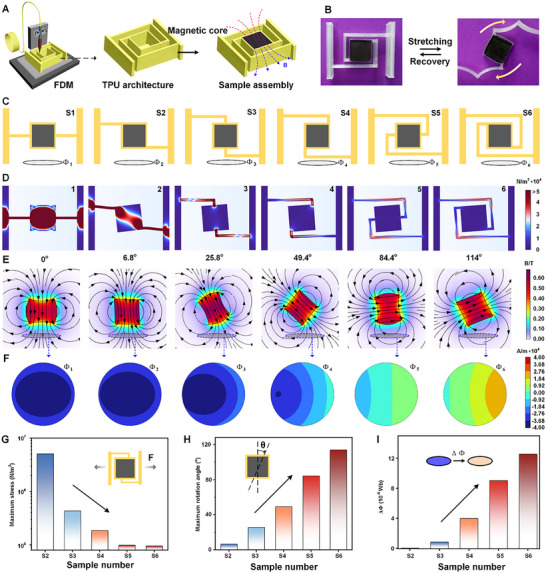
Design and testing of the stretchable, rotatable straight‐rod model (S‐series) for implementing the reversed magnetic field (RMF) strategy. A) RMFSs are prepared by 3D printing. B) Stretchable rotating samples printed using FDM. C) Straight‐rod models with different contact sites. D) Body stress distribution for all S‐series models stretched to the same distance. E) Magnetic field reversing phenomenon at maximum rotation for all S‐series models. F) Dynamic magnetic flux change in the coils as models are stretched to their maximum rotation. G) Maximum surface stress comparison for all models when stretched at the same distance. H) Maximum rotational angle for all models when stretched. I) Flux changes in the coil before and after stretching for all models at maximum rotation.

Current design approaches for magnetoelastic soft materials primarily achieve changes in magnetic flux through structural deformation, which restricts magnetic flux variation and hampers improvements in energy conversion efficiency. To address this, we designed reversed magnetic field samples (RMFSs). A series of stretching‐driven rotatable straight‐rod helical structures were fabricated using a fused deposition modeling (FDM) 3D printing method (**Figure** **2**A). The central hollow part was filled with magnetoelastic soft material composed of 90% (by weight) magnetic powder and 10% (by weight) Ecoflex, using the FDM process. An optical image of a representative sample is shown in Figure 2B. When a horizontal stretching force is applied, the magnetoelastic middle square easily rotates along the center due to the straight‐rod helical structure.

### 2.3 Simulation and Experimental Testing of RMFSs

To analyze the performance of the RMFSs, the samples were categorized as the S‐series based on the contact points between the straight‐rod helical structures and the magnetoelastic middle square (Figure 2C). The RMFSs consisted of models S1–S6, with S1 serving as the control (non‐rotatable) sample. The specific dimensions and optical images of all structures are provided in Figure S1 (Supporting Information).

Finite element analysis (FEA), including both mechanical and magnetic intensity simulations, was conducted on models S1‐S6. When the magnetoelastic middle squares of these models were stretched to a fixed distance of 2 mm, the simulation results showed a progressive decrease in the maximum surface stress from 5,092,700 N m^−2^ (S2) to 96,326 N m^−2^ (S6) (Figure 2G). This suggests a structure‐dominated energy consumption process. These results were further supported by the tensile force versus stretching distance curves for each model (Figure S2, Supporting Information). A color‐gradient analysis of the body stress distribution across the models (Figure 2D) indicated the force exerted during stretching. The maximum body stress decreased progressively from S1 to S6, which correlates with the increasing rotatability of the magnetoelastic middle squares from S1 to S6.

In model S1, the magnetic field direction remained fixed, and it required the highest force for deformation. In contrast, the maximum rotatable angle increased from S2 to S6, with the S6 model achieving a peak rotation of 114° while requiring just 5.87 N of force for deformation (Figure 2H).

The mechanically‐driven rotation of the magnetoelastic middle squares also resulted in significant changes in magnetic intensity, as analyzed through Maxwell analysis (Figure 2E). The magnetic flux changes in the coil beneath each sample were measured before and after stretching, as shown in Figure 2F. Details on the magnetic flux calculation are provided in Note S1 (Supporting Information). As the rotatable angle of the magnetoelastic middle squares increased from 6.8° (S2) to 114° (S6), the magnetic flux variation increased from 0.06 × 10^−6^ Wb (S2) to 12.55 × 10^−6^ Wb (S6) (Figure 2I). This result was corroborated by the magnitude of the magnetic flux density measurements in the coil (Figure S3, Supporting Information).

To further validate the simulation results, a 3D Gaussian scan was performed on the model, with the scan results presented in Figure S4A (Supporting Information). The scan results, depicted in Figure S4B (Supporting Information), showed the magnetic field intensity distribution along the sides of the magnetic block. The maximum magnetic field intensity observed in the scan was ≈60 mT, consistent with the maximum magnitude of magnetic flux density obtained in the simulation (Figure S3, Supporting Information), further validating the accuracy of the simulation.

### 2.4 Helical Structures Optimization

RMFSs with straight‐rod helical structures achieved a magnetic field rotation of up to 114°, corresponding to a 130% change in magnetic flux. The mechanoelectrical conversion of these RMFSs, both before and after stretching, can be calculated using Faraday's law of electromagnetic induction:

(1) $$E\left(V\right)=-\frac{n*\Delta \Phi }{\Delta t}$$

where E(V) is the output voltage, *n* is the number of rings, ΔΦ represents the total magnetic flux change, and Δ*t* is the response time of the RMFSs.

If the rotational angle of the magnetic field in RMFSs could reach 180°, the change ratio of total magnetic flux would increase up to 200%, leading to significantly enhanced electrical outputs. Unfortunately, RMFSs with straight‐rod helical structures failed to achieve this target, necessitating further optimization. Thus, smooth helical structures, such as Archimedean helix rotations and logarithmic spirals, were employed. From a structural mechanics perspective, smooth helical models unfold more effectively during stretching, enabling greater magnetic field rotation. In contrast, the straight‐rod model experiences stress concentrations at the corners during stretching, which limits the rotation and magnetic flux change (as visualized by the body stress distribution of the S6 model in Figure 2D).

**Figure 3:**
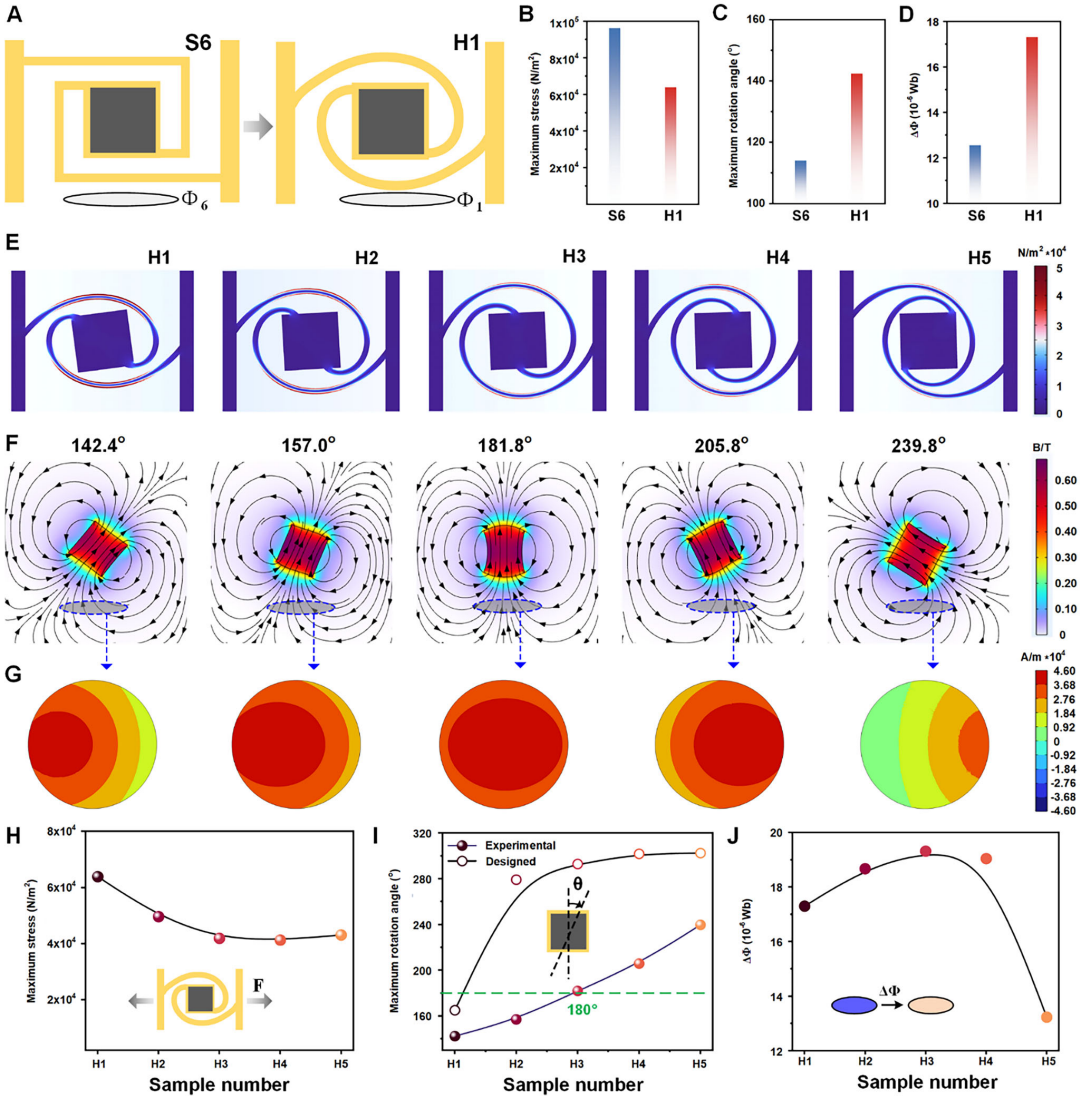
Optimization and testing of the helical structure RMF system (H‐series). A) Design of the Archimedean helix structure (design transition from linear to spiral models). B) Comparison of surface stress between H1 and S6 models when stretched at the same distance. C) Comparison of the maximum rotational angle between H1 and S6 when stretched at the same distance. D) Magnetic flux change comparison between H1 and S6 models before and after stretching. E) Body stress distribution for all helical models when stretched the same distance. F) Magnetic field reversing phenomenon at maximum rotation for all helical models. G) Dynamic magnetic flux change in the coil for all helical models at maximum rotation. H) Maximum surface stress comparison for all helical models when stretched at the same distance. I) Maximum rotational angle (purple line) compared to the designed rotational angle (black line) for all models. J) Magnetic flux change in the coil for all helical models at maximum rotation.

The Archimedean helix rotation model (H1) was introduced (**Figure** **3**A) with the governing equation for its shape:

(2) $$x\;=\left(a_{1}-b_{1}*t\right)\;*\cos\left(c_{1}*t\right),y\;=\left(a_{1}-b_{1}*t\right)\;*\sin\left(c_{1}*t\right)$$

where *a*_1_ = 5, *b*_1_ = 7.96, *c*_1_ = 0.53, and t_1_∈(0.3, 1.825*π).

Compared to the straight‐rod helical structure (S6), the Archimedean helix model exhibited lower surface stress during stretching (63,864 vs 96,326 N m^−2^, Figure 3B), resulting in a larger rotatable angle of the magnetoelastic middle square (142° vs 114°, Figure 3C). Consequently, the magnetic flux change increased from 12.55 × 10^−6^ Wb to 17.30 × 10^−6^ (Figure 3D).

However, despite these improvements, the Archimedean helix model's rotation angle was still limited, preventing the exponential increase in magnetic flux. Therefore, logarithmic spirals, known for their faster radius growth (Figure S5A, Supporting Information), were selected. Archimedean spirals are isometric spirals (red line in Figure S5A, Supporting Information), and their shape may not meet design requirements when larger pole angles are necessary. Therefore, a series of logarithmic spiral models (H2‐H5) were designed and fabricated. Detailed dimensions and optical images are provided in Figures S6 and S7 (Supporting Information). The governing equation for model H2 is:

(3) $$x\;=a_{2}\;\cos\left(s+c_{2}*t\right)*e^{b_{2}*t},\;y\;=a_{2}\;\sin\left(s+c_{2}*t\right)*e^{b_{2}*t}$$

where *a*_2_ = 8, *b*_2_ = 0.3, *c*_2_ = 0.79, s = 0.12, and t_2_∈(‐0.35*π, 1.565*π).

The control equations for H3‐H5 are:

(4) $$x\;=a\cos\left(t\right)*e^{b*t},\;y\;=a\sin\left(t\right)*e^{b*t}$$

where *a*_3_ = 7, *b*_3_ = 0.26, t_3_∈(0.7, 1.85*π); *a*_4_ = 9.5, *b*_4_ = 0.2, t_4_∈(0.089, 1.705*π); *a*_5_ = 9.9, *b*_5_ = 0.2, t_5_∈(0, 1.68*π).

Then, RMFSs with improved helical structural designs (H1–H5) underwent mechanical and magnetic simulation analyses. The mechanical simulation results revealed a general decreasing trend in the maximum surface stress, from 63,864 N m^−2^ (H1) to 43,095 N m^−2^ (H5) (Figure 3H). Body stress distribution plots are shown in Figure 3E. Compared to the straight rod models (S1‐S6), the improved helical models exhibited considerably reduced surface stress when stretched over the same distance. This indicates that the improved helical structures are more easily deformable.

Based on the experimental results, the maximum rotational angle for each model gradually increased from H1 to H5, with the spiral structure model reaching a maximum angle of 239.8° (purple line in Figure 3I). Notably, the H3 spiral structure model is capable of rotating beyond 180°. To elucidate the correlation between the maximum rotational angle and the designed structure, the equations for all models in the Cartesian coordinate system were transformed into polar coordinates, and the size of the angular interval for each model was subsequently calculated. The results are represented by the black line in Figure 3I. It was observed that a larger angular interval corresponds to a greater maximum rotational angle for the model. The maximum rotation angles of the models align with their designed rotation angles, showing a gradual increase from H1 to H5. The polar equations for H1 to H5 are as follows:

(5) $$r_{1}=a_{1}\;-\left(b_{1}/c_{1}\right)*\theta$$

(6) $$r_{2}=\frac{a_{2}}{e^{\left(s*b_{2}/c_{2}\right)}}\;*e^{\left(b_{2}/c_{2}\right)*\theta }$$

(7) $$r_{3}=a_{3}*e^{b_{3}*\theta }$$

(8) $$r_{4}=a_{4}*e^{b_{4}*\theta }$$

(9) $$r_{5}=a_{5}*e^{b_{5}*\theta }$$

Furthermore, the shape of each helical structure was determined using these polar coordinate equations (Figure S5B, Supporting Information). The design concept is further validated by the helical shapes produced.

Meanwhile, the magnetic intensity distributions of RMFSs (H1–H5) during stretching were analyzed (Figure 3F), and the magnetic flux change in the coil of each model after stretching was assessed (Figure 3G). The magnetic simulation results (Figure S8, Supporting Information) indicate that the magnetic flux change initially increases and then decreases from H1 to H5, with H3 exhibiting the highest flux change value of 19.3 × 10^−6^ Wb (Figure 3J). The initial magnetic flux, calculated at 9.6 × 10^−6^ Wb when all models are in the non‐rotated state, shows that the magnetic flux change in the H3 model becomes twice the initial flux.

The exponential increase in magnetic flux is primarily attributed to the 180° rotation angle and magnetoelastic deformation, which is consistent with the theoretical framework discussed earlier. Specifically, when the H3 model is rotated by 180°, the magnetic flux changes from Ф_0_ (initial flux) to ‐Ф_0_, leading to the maximum magnetic flux change. Moreover, when the angle of rotation is maximized. That is, after the model is fully unfolded, the magnetoelastic soft material is stretched to the point of deformation, which further increases the change in magnetic flux. In contrast, rotation angles either greater or less than 180° do not result in a magnetic flux change equal to twice the initial value.

### 2.5 Mechanoelectrical Conversion Performances of RMFSs

**Figure 4:**
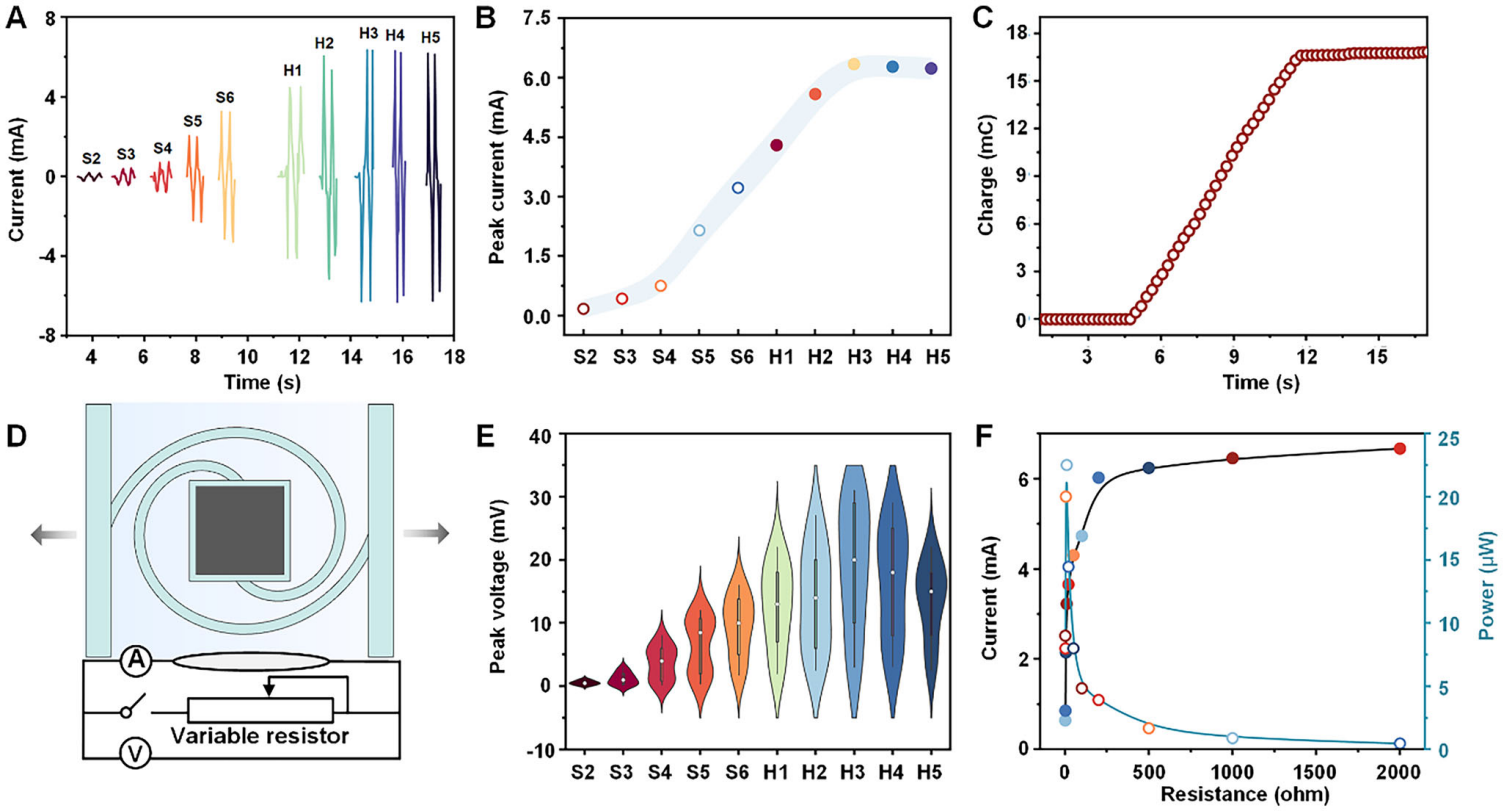
Electrical performance analysis of all RMFSs. A) Current signals comparison for all RMFSs. B) Peak current values for all RMFSs (*n*  =  3, data presented as mean ± s. d.). C) Coulomb integral curve for the current signal of the H3 model. D) Circuit diagram with external load resistance ranging from 1 to 2000 ohms. E) Violin plot of peak voltage signals for all RMFSs under different external loads. F) Current and power curve for the H3 model under varying external load resistance.

The mechanoelectrical conversion performances of the RMFSs, including both the straight‐rod models (S2‐S6) and the improved helical models (H1–H5), were evaluated by connecting each system to a uniform testing setup (Figure S9, Supporting Information). The recorded current signals over time are shown in **Figure** **4**A. For the straight‐rod models, the current signals increase progressively from S2 to S6. In contrast, for the helical models, the current initially rises, peaking at 6.34 mA in the H3 model, before slightly decreasing in the later models (Figure 4B). Overall, the helical models demonstrated significantly stronger current outputs compared to the straight‐rod models, a trend corroborated by the peak voltage signals (Figure S10, Supporting Information), which align well with the magnetic simulation results (Figures 2I and 3J). Among the models, H3 exhibited the best performance. To further assess its capabilities, Coulomb integration of the current signal was performed, revealing a Coulomb aggregative value of ≈17 mC (Figure 4C), which is substantially higher than those reported in previous studies.^[^ ^14^, ^15^ ^]^

Further testing involved connecting each RMFS (S and H series) to an external load circuit with resistances ranging from 1 to 2000 ohms (Figure 4D). Violin plots of the voltage peaks for each model under varying loads are displayed in Figure 4E. Once again, the H3 model proved to be the most effective, achieving a peak current of 6.67 mA at a load resistance of 2000 ohms. Peak currents for different resistances in the H3 model's circuit were calculated, and the corresponding power output was plotted (Figure 4F). The H3 model achieved its highest power output of 22.5 µW at an external load resistance of 10 ohms. At an external load of 1000 ohms, the power density reached 1.5 mW m^−2^, which is 10^3^ times higher than current magnetoelastic soft materials.^[^ ^12d^ ^]^

### 2.6 Evolution of Helical Model for RMFSs

While the mechanoelectrical outputs of RMFSs already surpass most reported values in the literature, further performance enhancements can be achieved by reducing the distance between the magnetoelastic middle and the underlying coil. In the original models, the magnetoelastic middle had a square shape, and its rotation during stretching caused variations in the distance between the magnetoelastic surface and the coil. To address this issue, the shape of the magnetoelastic middle was modified from a square to a circular design (Figure S11A, Supporting Information). This change ensures that the distance between the nearest point of the magnetoelastic cylinder and the coil remains constant, allowing for a significant reduction in the distance from the original 15 to 0.5 mm, which could greatly enhance power generation.

Building on this concept, an improved model series, termed the R‐series, was designed. Various RMFSs were fabricated with different distances (denoted as Δ*h*) between the magnetoelastic circle, ranging from 15.5 to 0.5 mm. These models were named R, followed by the respective distance.

When each RMFS was connected to the testing system, the peak current increased as the distance Δh decreased. At Δh = 0.5 mm, the peak current value reached 43 mA (Figure S11B, Supporting Information), setting a record for power generation. Ultimately, the R0.5 model achieved a power output of 8.6 mW (Figure S11C, Supporting Information). This represents a significant enhancement over current reports, with detailed data provided in Table S2 (Supporting Information).

A comparison between the H3 and R0.5 models (Figure S11D,E, Supporting Information) revealed that the R0.5 model achieved a record‐breaking current density of 7.17 mA cm^−2^, which is 2.4 times higher than previous reports that are based solely on deformation‐induced magnetic field variation^[^ ^10^, ^12^, ^16^ ^]^ (Figure S11F and Table S2, Supporting Information). In addition, this work was compared with some representative studies in the fields of triboelectric, piezoelectric, and moisture‐driven energy harvesting. The results of the comparison indicate that the current density achieved in this study has reached a remarkably high level (Figure S11F, Supporting Information). This demonstrates the remarkable potential of the optimized circular design for significantly improving the energy conversion efficiency of RMFSs.

### 2.7 Applications of RMFSs

**Figure 5:**
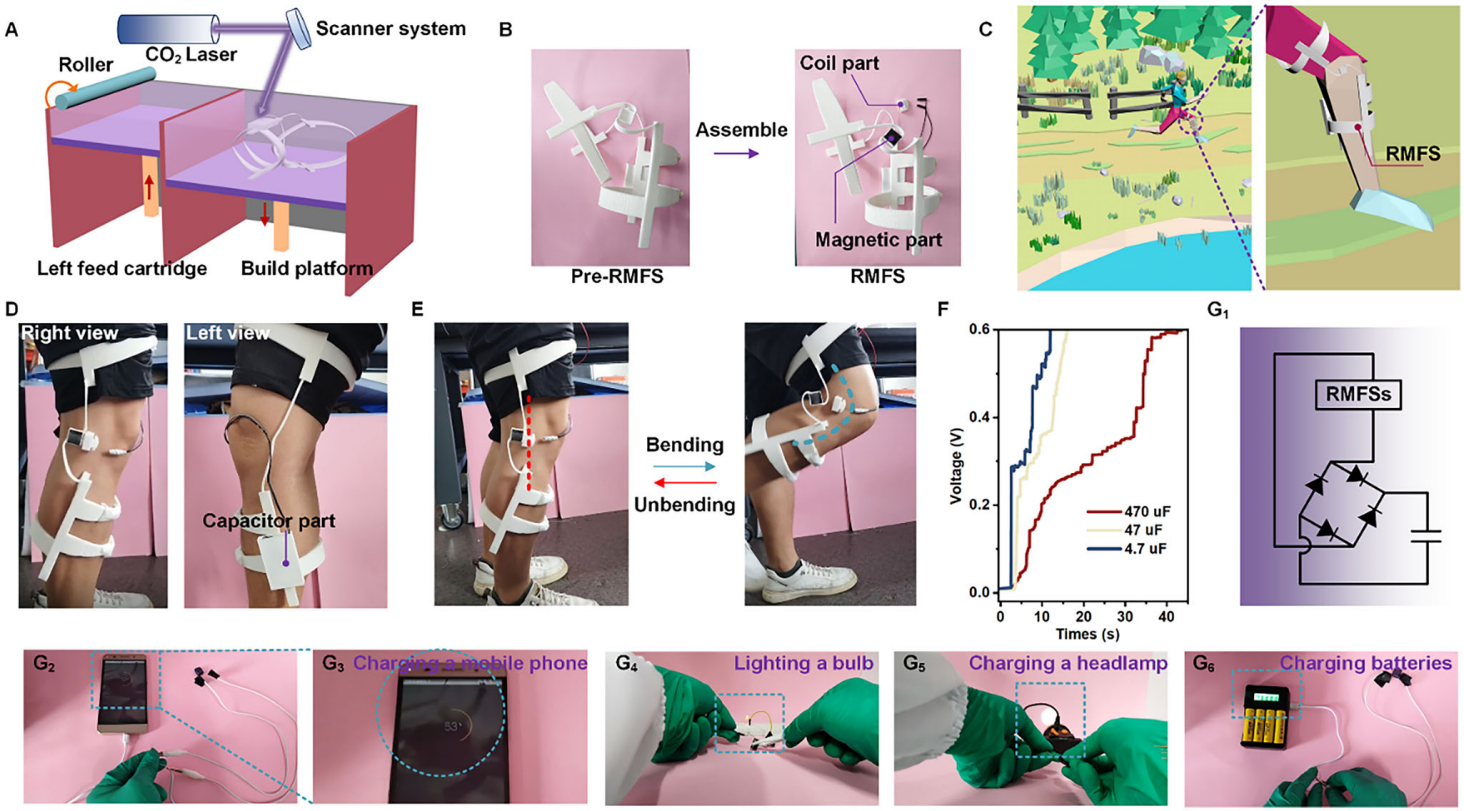
Wearable energy harvesting with a giant current output. A,B) A wearable knee pad using the reversed magnetic field strategy with a helical structure was designed and 3D printed using SLS technology. C) The printed models are applied in outdoor scenarios for self‐powered energy harvesting. D) Left and right views of the self‐powered system worn on the knee. E) Schematic showing the stretching and recovery cycle of the self‐powered system. F) Leg movement drives the helical structure to rotate, generating cyclic motion that charges three capacitors (470, 47, and 4.7 µF). Final voltage curves are shown. (G_1_) Schematic diagram of charging a capacitor using the helical structure with magnetic field reversal. (G_2_)–(G_6_) The electrical energy generated by the leg's reciprocating motion is stored in a capacitor, which is then used to power various electronic devices, including a smartphone, LED light‐emitting diode, headlamp, and rechargeable batteries.

Wearable devices have a broad development space in medical, sports, robotics, and other fields.^[^ ^17^ ^]^ Here, the H3 model of RMFSs was integrated into a wearable legging system. The connective components were 3D‐printed using flexible thermoplastic polyurethane via selective laser sintering (SLS) (**Figure** **5**A). The magnetoelastic middle and a capacitor were placed on either side, resulting in a wearable knee pad (Figure 5B). When worn on the knee (Figure 5C,D), biomechanical movements, such as bending and stretching of the leg, are harnessed to generate electricity, which is stored in the capacitor for further use. Although lithium batteries can also be used as an energy source for outdoor activities,^[^ ^18^ ^]^ their functionality is significantly limited once depleted. Therefore, a solution combining spiral structures with wearable electronic devices is proposed. Using 3D printing technology, a kneepad is fabricated. This approach enables the conversion of mechanical energy generated by human motion, such as walking or running, into electrical energy to power electronic devices. This method is crucial for ensuring energy security during outdoor survival and activities.

Experimental results demonstrated the device's capability to charge capacitors of 470, 47, and 4.7 µF to a voltage of 0.6 V within 43, 16, and 12 s, respectively (Figure 5E,F). Once charged to 3 and 5 V, these capacitors were able to power smartphones, LED light‐emitting diodes, outdoor headlamps, and rechargeable batteries (Figure 5G_1_‐G_6_; Videos S1–S4, Supporting Information), ensuring consistent and reliable operation.

Additionally, the durability and long‐term stability of the printed structure were evaluated. Taking H3 model as an example, the model was stretched 10 000 times, and the force, displacement, and electrical signal responses over time were recorded (as shown in Figure S12, Supporting Information). Results demonstrate that during 10 000 stretching cycles, the force and displacement responses over time exhibited excellent stability (Figure S12A,B,D,E, Supporting Information). Simultaneously, the electrical signal responses over time were also assessed during the stretching process (Figure S12C,F, Supporting Information), revealing that the electrical signal remained highly stable even under frequent stretching.

Considering the practical application requirements of wearable electronics, we tested the electrical signal responses of the helical structure over time at different temperatures (15–30 °C) and evaluated its performance in rainy conditions (Figure S13, Supporting Information). The results indicate that in different temperature conditions, there was a slight increase in the intensity of the generated electrical signals with rising temperatures. Overall, the electrical signal intensity exhibited only minimal improvement with temperature increases, and the rainy environment had a negligible effect on the output of the electrical signals.

## 3 Conclusion

This work presents a novel design strategy for achieving a reversed magnetic field to increase the force‐induced magnetic field variation using a helical structure. Through simulations and experimental testing, it was shown that the logarithmic helical structure could achieve a 200% change in magnetic flux, with a peak current of 6.34 mA. Further structural optimization led to a peak current of 43 mA and a power output of 8.6 mW, along with a record‐breaking current density of 7.17 mA cm^−2^—an improvement of 2.4‐fold over previous reports that rely solely on deformation‐induced magnetoelasticity. The optimized logarithmic helical structure was then integrated into a wearable energy harvesting system. This system successfully converted biomechanical energy into electrical energy, stored in capacitors, and used to power smartphones, LEDs, headlamps, rechargeable batteries, and other electronic devices. The design strategy proposed here offers new insights and potential for the application of magnetoelastic soft materials in energy harvesting and wearable electronics.

## 4 Experimental Section

### Materials

NdFeB powder (400 mesh) was purchased from Guangzhou Xinnuode Transmission Parts Co., Ltd., and two‐component liquid silicone (Ecoflex, T600#A&B) from Shenzhen Hong Ye Jie Technology Co., Ltd. Coils were custom‐made. Thermoplastic polyurethane (TPU) filament for FDM was sourced from Zhuhai Sanlu Technology Co., Ltd., while the standard resin used for liquid‐crystal display (LCD) printing was purchased from Shenzhen Esun Industrial Co., Ltd. TPU powder for SLS was obtained from Beijing Huanze New Material Technology Co., Ltd.

### Fabrication of RMFSs

First, S‐series and H‐series models were printed using an FDM printer (CR‐2020, Shenzhen Creality 3D Technology Co., Ltd.). Liquid silicone components A and B (Ecoflex) were mixed in a 1:1 mass ratio. NdFeB magnetic powder was then combined with the mixed Ecoflex in a 90:10 weight ratio, forming a dark, viscous mixture. This mixture was poured into the 3D‐printed models and cured at 60 °C for 2 h. After solidification, all models were magnetized vertically at 1800 V using a magnetizer (MA‐2030, Shenzhen Jiuju Industrial Equipment Co., Ltd.). The R‐series models were printed using LCD technology, with curing and magnetization procedures identical to the S‐ and H‐series.

### Fabrication of Wearable Helical Model

The wearable helical model was printed via SLS, with magnetic powder curing and magnetization handled as described for the other RMFSs.

### Numerical Simulation

FEA simulations were conducted using COMSOL Multiphysics 6.2, with the modeling process also facilitated by this software. The simulation was divided into two parts: mechanical and magnetic. All RMFS models were stretched by 2 mm under identical conditions, with tensile force applied to plane T (Figures S1 and S6, Supporting Information). The results were used to analyze the distribution of body stresses and calculate the maximum surface stresses on the models. This analysis helped compare the ease of tensile deformation across the models and verify the maximum achievable rotation angle for each. Additionally, energy consumption during the stretching process was qualitatively analyzed for each model. A coil of equal radius was used to simulate the magnetic behavior. Each model was stretched to its maximum angle, and the change in magnetic flux within the coil was calculated by comparing flux values before and after stretching, with the remanent magnetization (Br)​ set to 0.8 T. Furthermore, the magnetic field intensity and flux density within the coil were calculated to dynamically visualize and analyze changes in the magnetic field throughout the stretching process.

### Characterization

Optical images and videos of the sample and measurement processes were captured using a commercial digital camera (α6300, Sony). A 3D Gauss meter was employed to measure the spatial magnetic field distribution of the models. The scanning planes were L‐planes (Figures S1 and S6, Supporting Information), with the probes positioned 15 mm from the sides of the magnetic blocks. This instrument was provided by Beijing Cuihai Jiacheng Magnetoelectric Technology Co., Ltd. Each model was stretched at a uniform speed of 10 cm s^−1^, and the current signals were recorded using a DMM7510 voltmeter (Tektronix Technologies Co., China).

### Statistical Analysis

Data are presented as mean ± s. d., with a sample size of *n* = 3 for each statistical analysis.

The participant (X.C.) shown in Figure 5D,E provided informed consent for his participation and the publication of the data and images.

## Conflict of Interest

The authors declare no conflict of interest.

## Author Contributions

B.S. contributed to the initial concept. J.C. provided professional guidance. L.Y. proposed specific structural design ideas. X.C. performed all experiments and simulations. D.T. constructed all the models. Y.X. and Z.L. assisted with simulations. M.C., P.C., Y.L., and S.Z. provided support during the performance testing of the models. X.C., F.M., J.C., and B.S. analyzed the data. X.C. wrote the manuscript, with B.S. and J.C. offering writing guidance. All authors reviewed and commented on the manuscript.

## Supporting information



Supporting Information

Supplemental Video 1

Supplemental Video 2

Supplemental Video 3

Supplemental Video 4

## Data Availability

The data that support the findings of this study are available in the supplementary material of this article.
