# Supplementary material for: Logarithmic Helical Design for Reversed Magnetic Field in Magnetoelastic Soft Matters with Giant Current Outputs
Source: Adv Sci (Weinh). 2025 May 11;12(28):2505157. doi: 10.1002/advs.202505157 (PMC12302534; doi:10.1002/advs.202505157)
Supplement: Supplementary file 1 — Supporting Information [file ADVS-12-2505157-s003.docx]

Supporting information for

**Logarithmic Helical Design for Reversed Magnetic Field in Magnetoelastic Soft Matters with Giant Current Outputs**

Xiaojun Chen, Farid Manshaii, Dianyu Tang, Yizhuo Xu, Zhuofan Li, Manhui Chen, Peng Chen, Yike Li, Shanfei Zhang, Lei Yang*, Jun Chen*, Bin Su*

**Content**

**Supporting Figures**

Figure S1. Dimensions and actual photographs of models S1 to S6.

Figure S2. The relationship between tension force and stretching distance for models S2 to S6.

Figure S3. Process of changing the magnitude of magnetic flux density for models S1 to S6.

Figure S4. 3D Gaussian scanning of S-series models.

Figure S5. Polar coordinate plots for models H1 to H5.

Figure S6. Dimensions and actual photographs of models H1 to H5.

Figure S7. Schematic of model stretching.

Figure S8. Process of changing the magnitude of magnetic flux density for models H1 to H5.

Figure S9. Circuit diagram for measuring the electrical signals of RMFSs, including both S-series and H-series models.

Figure S10. Plots of peak voltage signals for all models.

Figure S11. Improved performance of R-series models.

Figure S12. Durability and stability evaluations of H3 model.

Figure S13. Time-response curves of electrical signals for H3 model under different temperatures and rainy conditions.

**Supporting Tables**

Table S1. Detailed indications of the rate of magnetic flux change for representative works.

Table S2. Detailed indications of current peaks and densities for representative works.

**Supporting Notes**

Note S1. Detailed magnetic flux calculations for all models.

**Supporting Videos**

Video S1. Charging a smartphone using power stored in a capacitor (470 F) through the knee’s helical structure.

Video S2. Charging an LED light using power stored in a capacitor (470 μF) through the knee’s helical structure.

Video S3. Charging a field headlamp using power stored in a capacitor (470 μF) through the knee’s helical structure.

Video S4. Charging rechargeable batteries using power stored in a capacitor (470 F) through the knee’s helical structure.


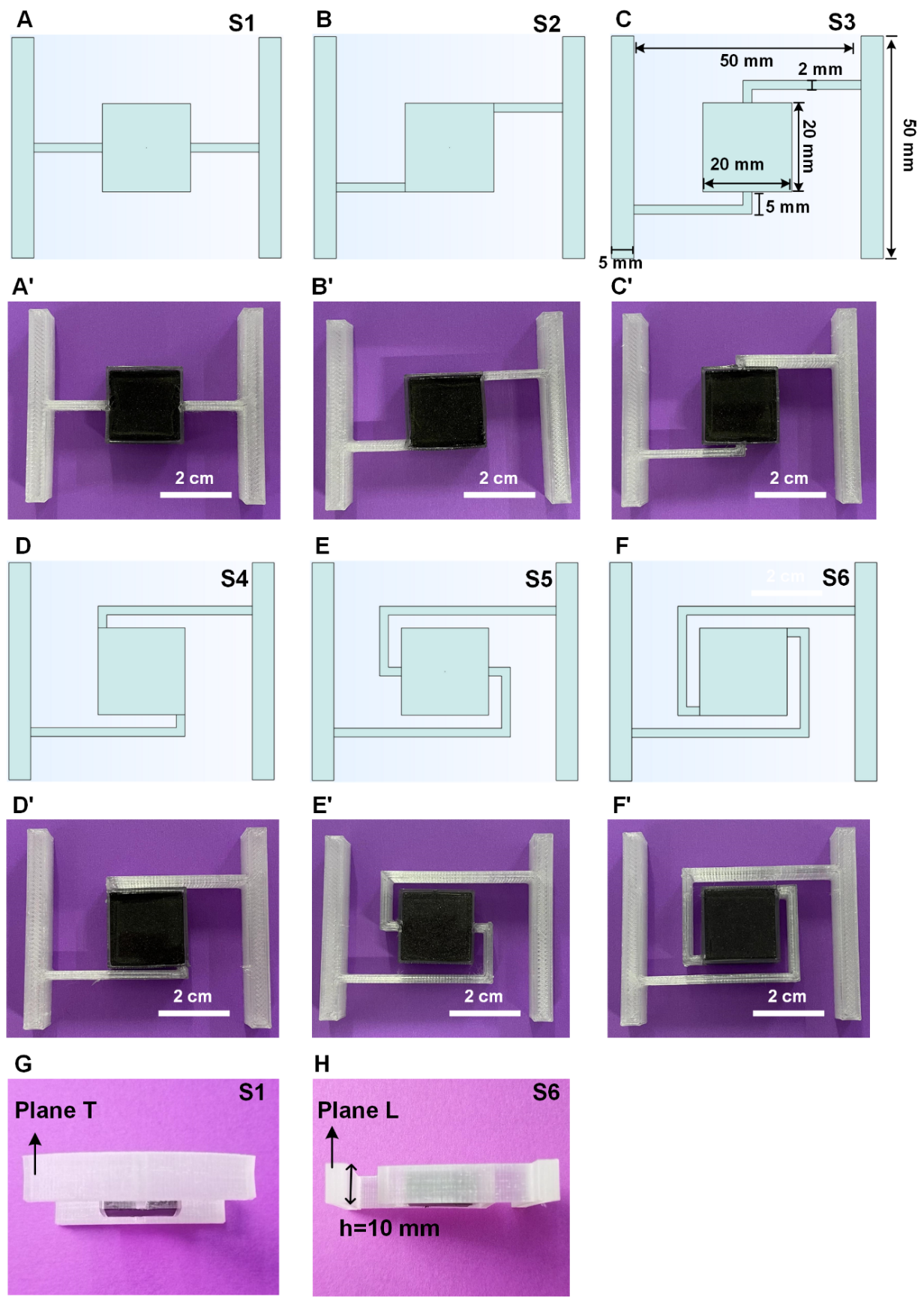


**Figure S1.** Dimensions and actual photographs of models S1 to S6. (A)-(F), Design drawings and dimensions of all models. (A')-(F'), actual photographs of all models. G, top view of model S1, indicating the surface where force is applied. H, top view of model S6, showing the thickness of the S-series and the 3D Gaussian scanning plane.


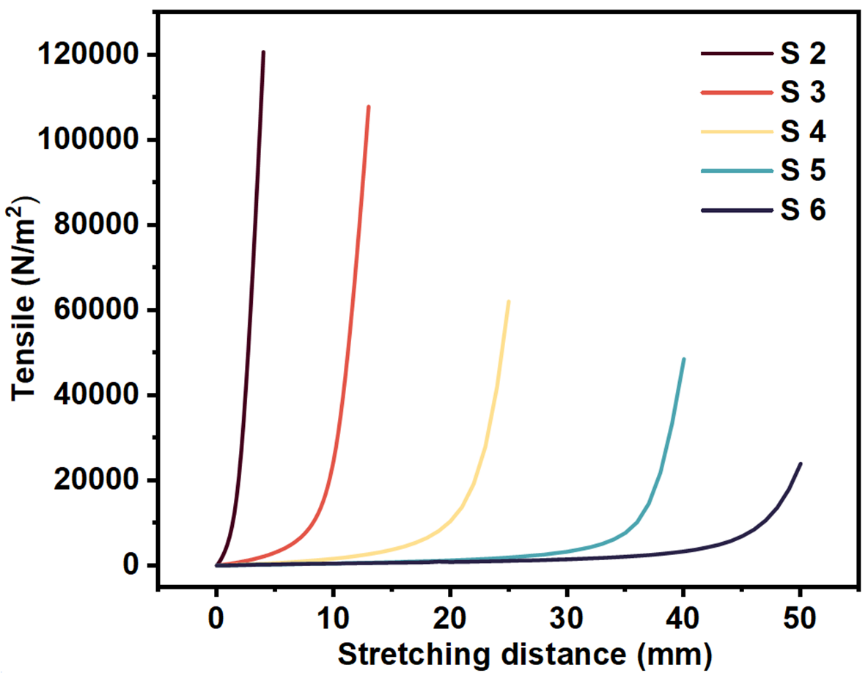


**Figure S2.** The relationship between tension force and stretching distance for models S2 to S6.


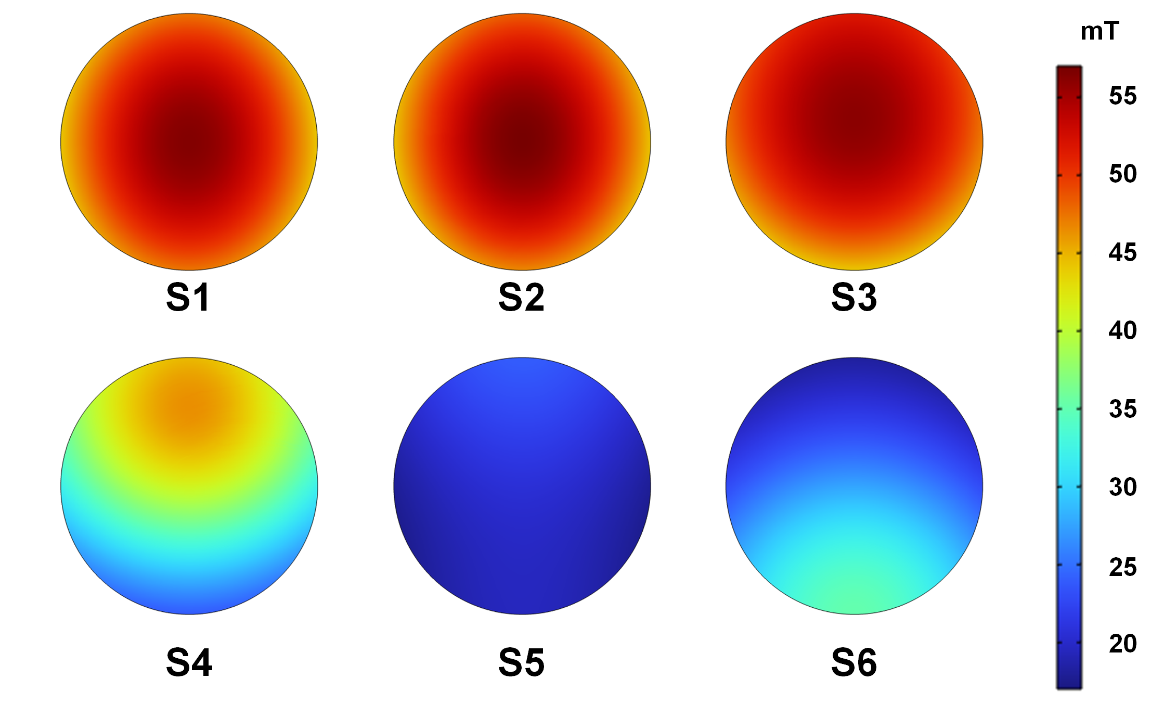


**Figure S3.** Process of changing the magnitude of magnetic flux density for models S1 to S6. Show the magnitude of magnetic flux density in the coil when each model is stretched to its maximum angle.


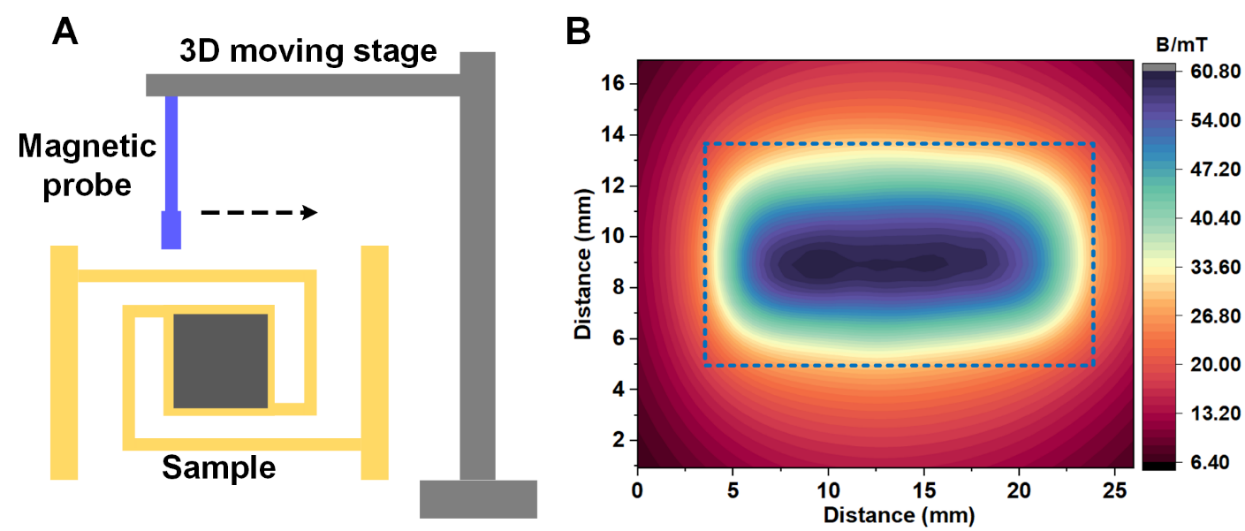


**Figure S4.** 3D Gaussian scanning of S-series models. (A), (B), schematic and data results (side scanning of the magnetic block), respectively.


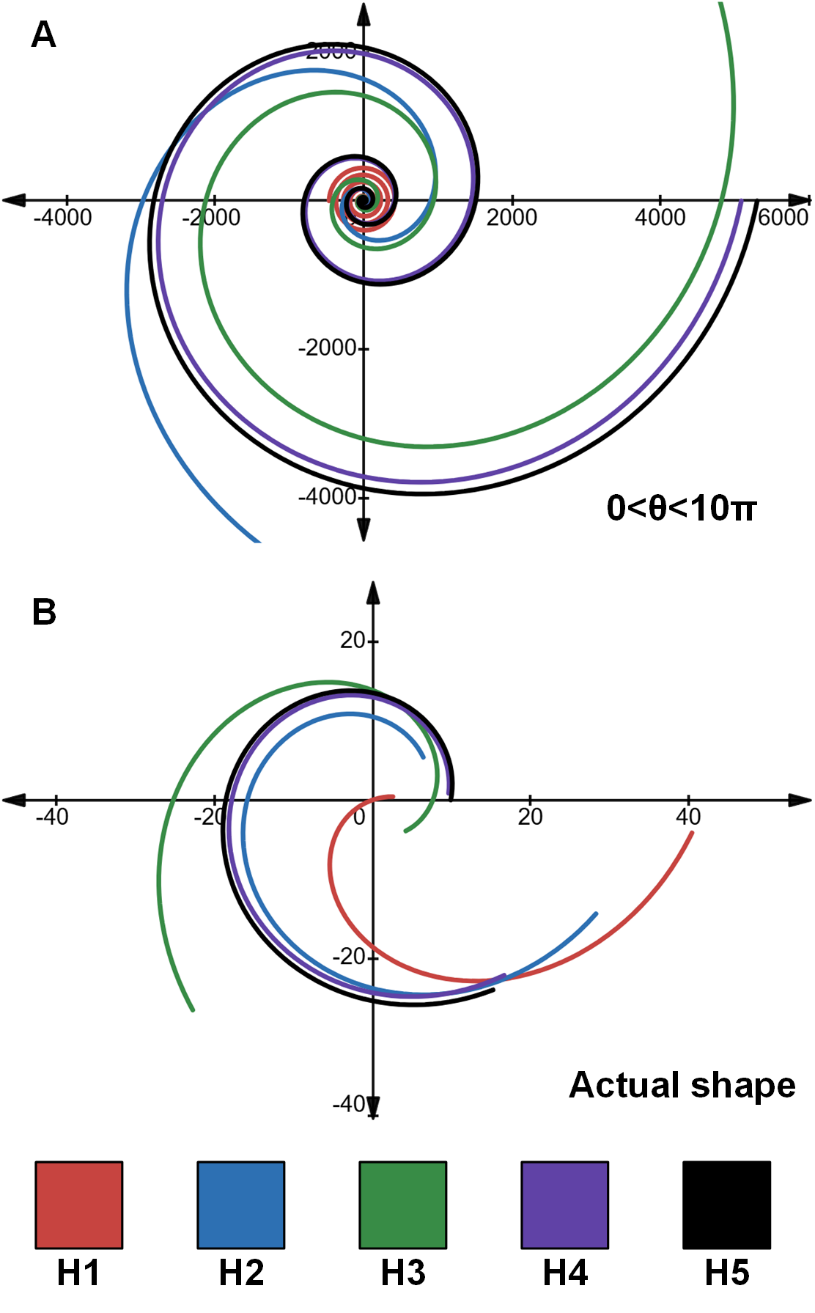


**Figure S5**. Polar coordinate plots for models H1 to H5. (A) plot with the same range of values (0<θ<10π). (B) plot with the actual range of values.

**
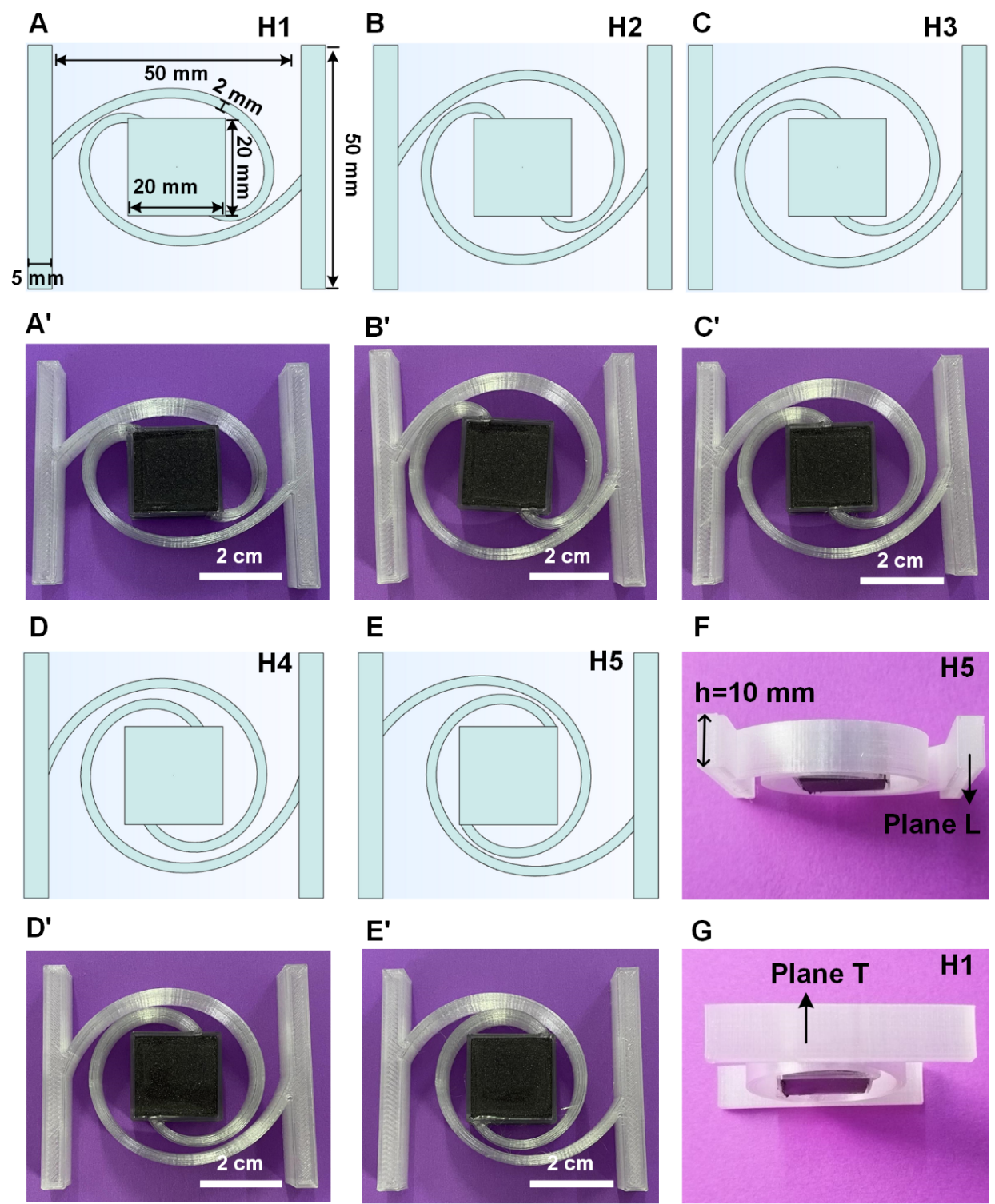
**

**Figure S6**. Dimensions and actual photographs of models H1 to H5. (A)-(E) Design drawings and dimensions of all models. (A')-(E') actual photographs of all models. F, top view of model H5, showing the thickness of the H-series and the 3D Gaussian scanning plane. G, top view of model H1, indicating the surface where force is applied.

**
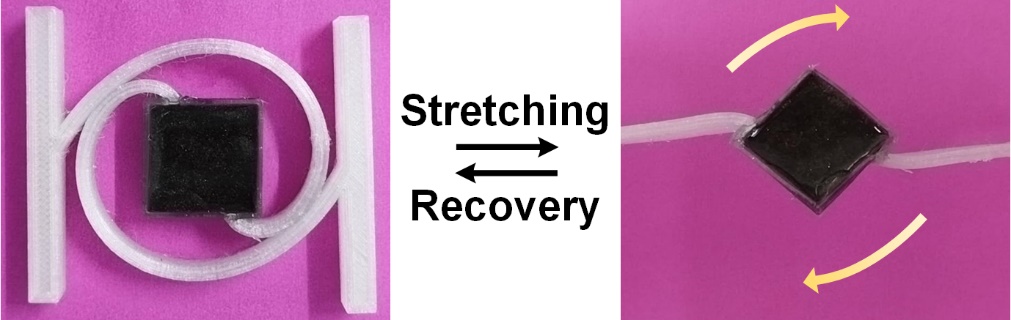
**

**Figure S7.** Schematic of model stretching.


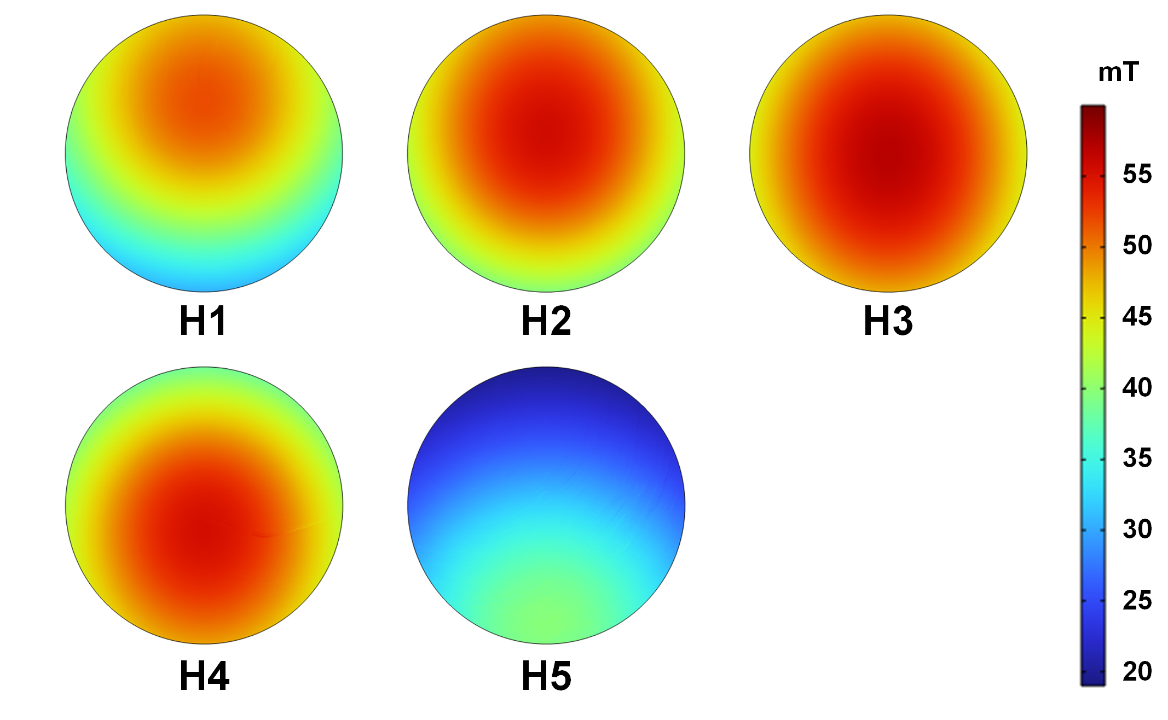


**Figure S8.** Process of changing the magnitude of magnetic flux density for models H1 to H5. Show the magnitude of magnetic flux density in the coil when each model is stretched to its maximum angle.


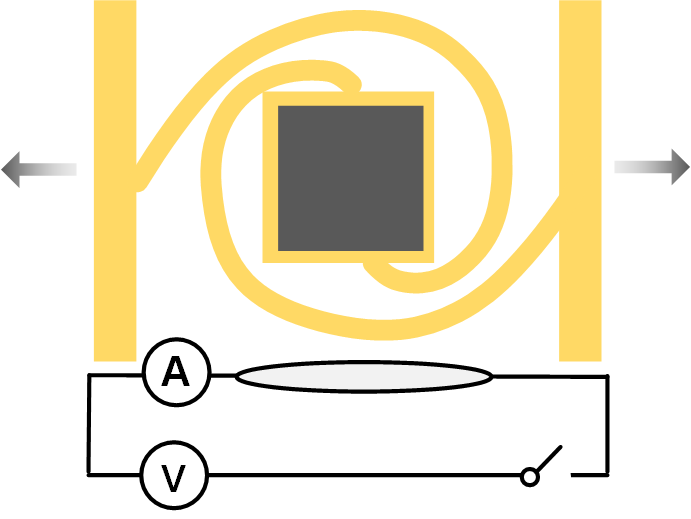


**Figure S9.** Circuit diagram for measuring the electrical signals of RMFSs, including both S-series and H-series models.


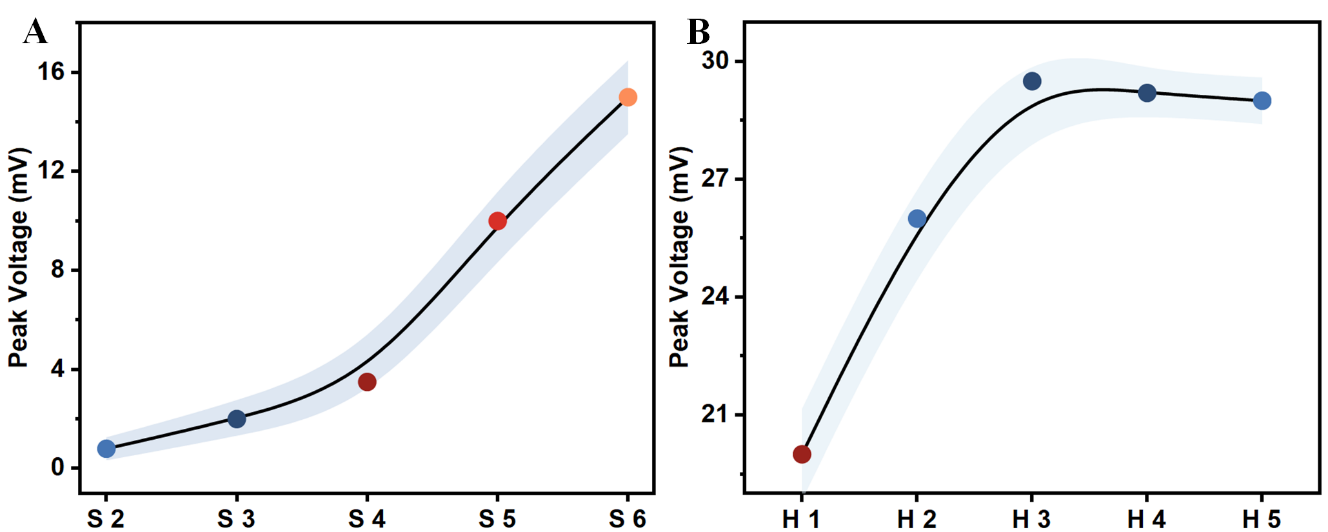


**Figure S10.** Plots of peak voltage signals for all models. (A) voltage peak signals for the S-series models. (B) voltage peak signals for the H-series models.


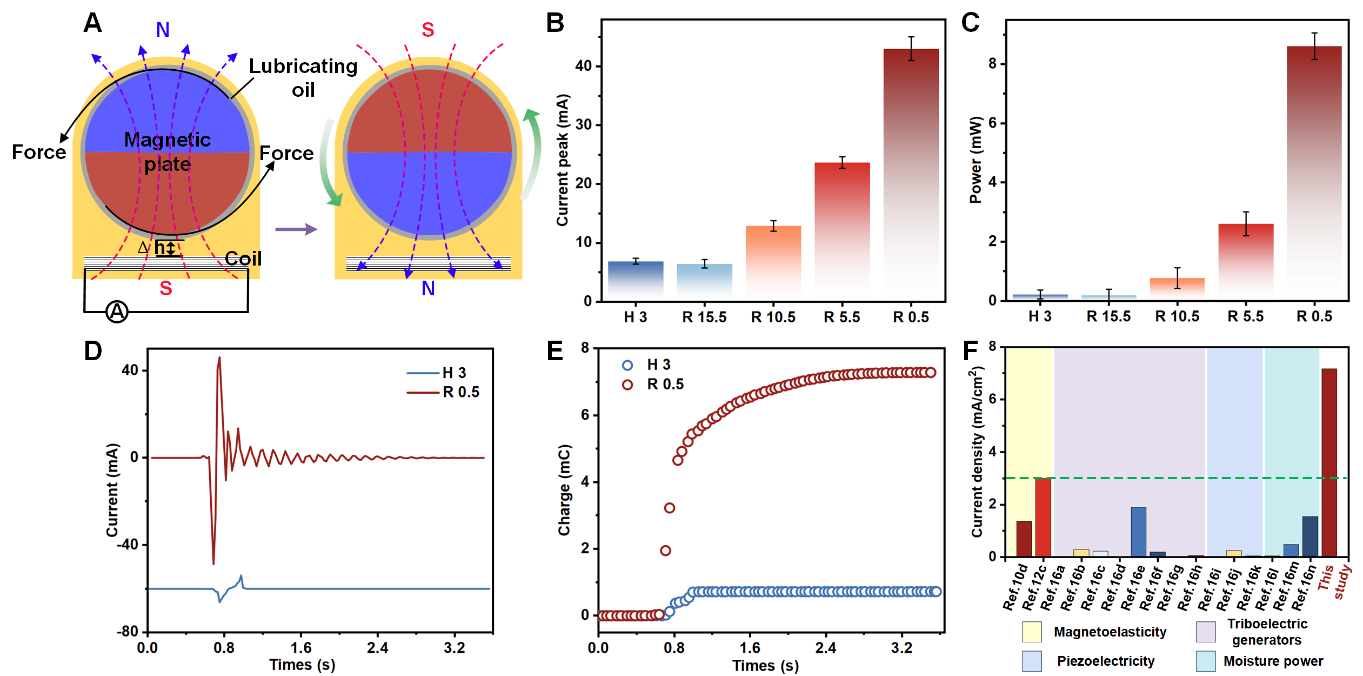


**Figure S11.** Improved performance of R-series models. (A) schematic diagram for measuring the electrical signals of the R-series models. The central magnetic block can rapidly rotate under the tensile force applied to both sides, causing the magnetic field to reverse. (B) current peak signals for R-series models and model H3. (C) power diagram comparing R-series models and model H3. (D) comparison of current signals between model R0.5 and model H3. (E) comparison of Coulomb integral curves between model R0.5 and model H3. (F) current densities for various representative works in the field of magnetoelectricity.


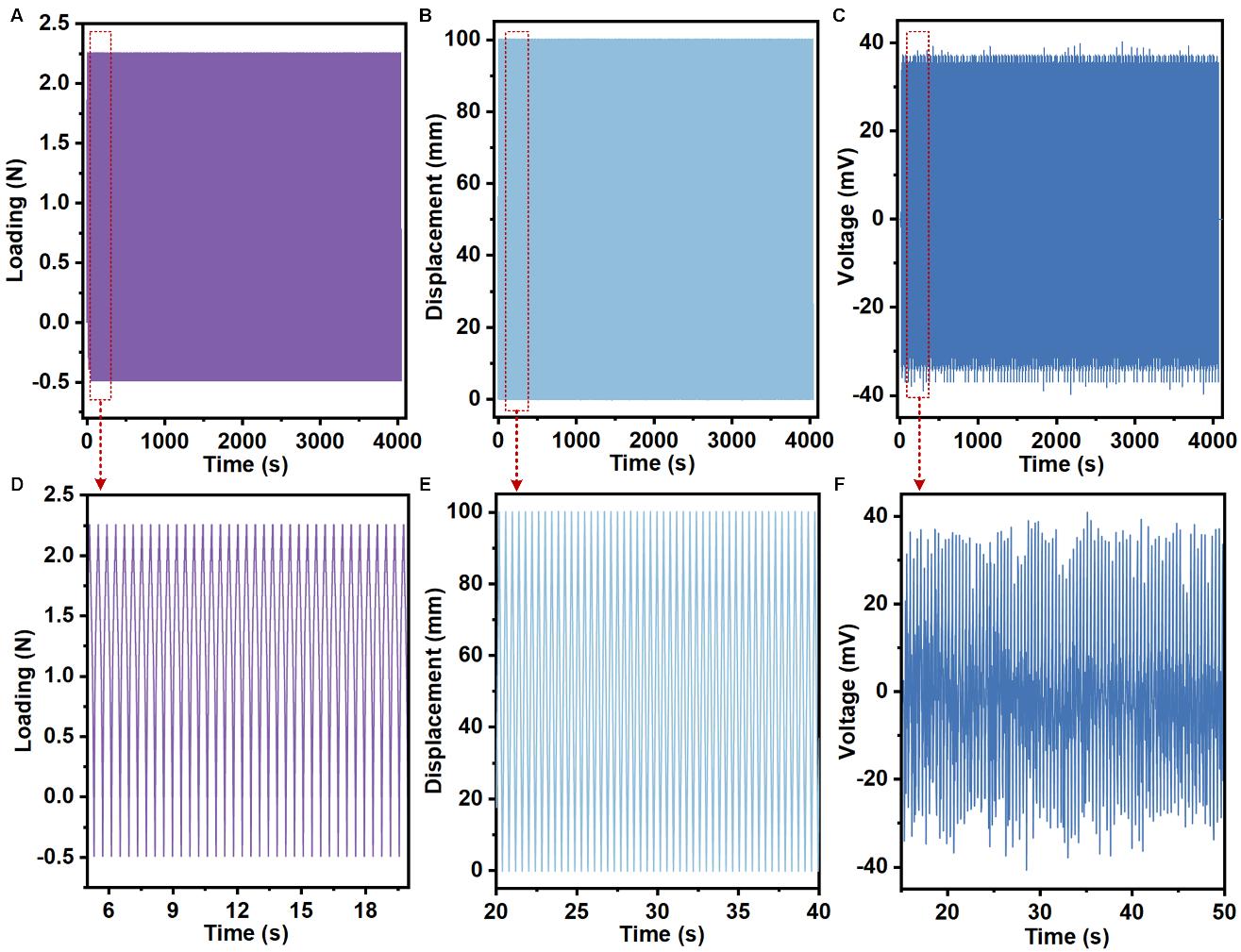


**Figure S12.** Durability and stability evaluations of the H3 model. (A) Force versus time curve; (B) Tensile displacement versus time curve; (C) Voltage signal versus time curve; (D), (E), and (F) correspond to partial time segments of (A), (B), and (C), respectively.


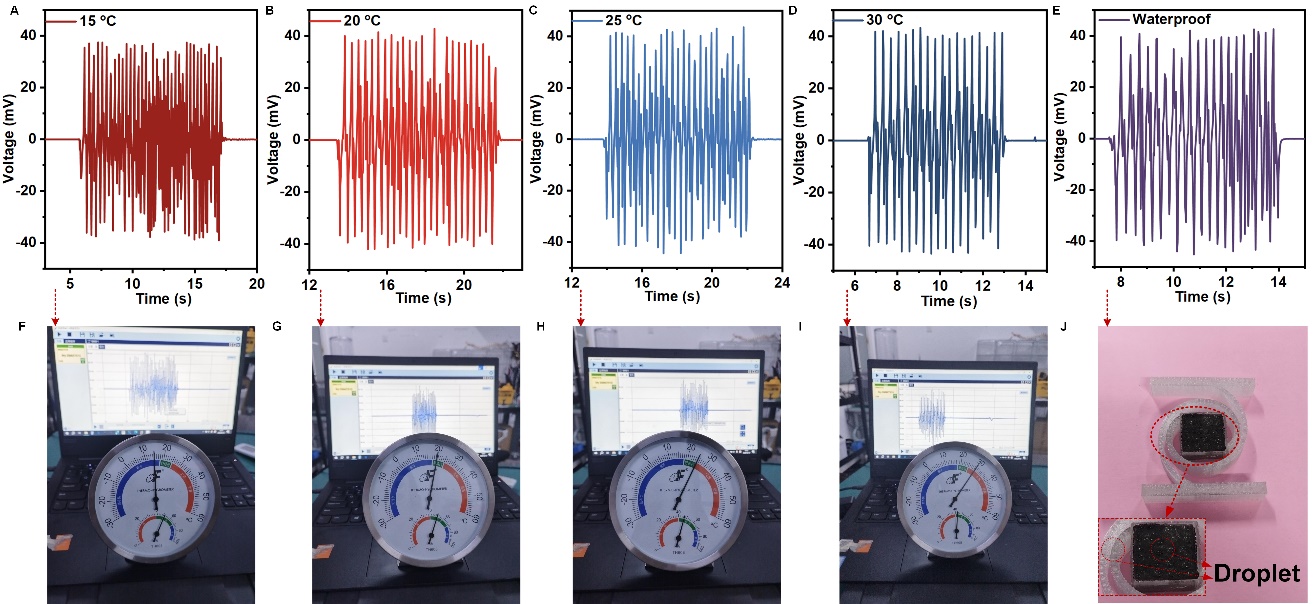


**Figure S13**. Time-response curves of electrical signals for the H3 model under different temperatures and rainy conditions. (A)-(D) Electrical signal vs. time curves for the H3 model at different temperatures (15 ^o^C, 20 ^o^C, 25 ^o^C, and 30 ^o^C); (F)-(I) corresponding testing environments for (A)-(D) with temperature displayed; (E) Electrical signal vs. time curve with water droplets present on the model’s surface; (J) Details of the model surface under wet conditions.

**Table S1.** Detailed indications of the rate of magnetic flux change for representative works.

| **Reference Number** | **ΔΦ/Φ_0_** |
| --- | --- |
| **REF.1** | **0.43** |
| **REF.2** | **0.50** |
| **REF.3** | **0.33** |
| **REF.4** | **0.75** |
| **REF.5** | **0.80** |
| **REF.6** | **0.46** |
| **REF.7** | **0.15** |
| **REF.8** | **0.77** |
| **REF.9** | **0.35** |
| **REF.10** | **0.29** |
| **This work** | **2.00** |

**Table S2.** Detailed indications of current peaks and densities for representative works.

| **Reference Number** | **Current Peak (mA)** | **Current Density (mA/cm^2^)** |
| --- | --- | --- |
| **REF.4** | 12.4 | 1.37 |
| **REF.11** | 24.63 | 2.99 |
| **REF.12** | 0.24 | 0.03 |
| **REF.13** | 0.32 | 0.29 |
| **REF.14** | 0.35 | 0.02 |
| **REF.15** | 1.95 | 0.05 |
| **REF.16** | 0.17 | 0.48 |
| **REF.17** | 5 | 0.22 |
| **REF.18** | N/A | 0.25 |
| **REF.19** | N/A | 0.02 |
| **REF.20** | 1.7 | 1.55 |
| **REF.21** | N/A | 1.90 |
| **REF.22** | N/A | 0.20 |
| **REF.23** | 0.012 | 0.03 |
| **REF.24** | N/A | 0.07 |
| **REF.25** | N/A | 0.05 |
| **This work** | **43** | **7.17** |

**Note S1.** Detailed magnetic flux calculations for all models.

The magnetic flux in the coil can be calculated for all models when they are stretched. The residual magnetism (B_r_) was set at 0.8 T.

The copper coil is modeled as a series of parallel rings with the same horizontal diameter under the following conditions:

1) The equivalent ring is coaxial.

2) The coil and its simplified equivalent model are assumed to exist in an infinite vacuum.

According to Faraday’s law of electromagnetic induction:

$E=-\frac{\Delta\Phi}{\Delta t}$ **(S1)**

Where $E$ is the induced electromotive force, $\Delta\Phi$ is the total change in magnetic flux for one turn of the coil, and $\Delta t$ is the time interval during which the coil is stretched.

$\Delta\Phi=\Phi_{after}-\Phi_{before}$ **(S2)**

$\Delta t=t_{after}-t_{before}$ **(S3)**

Here, $\Phi_{after}$ represents the total magnetic flux after the coil is stretched, and $\Phi_{before}$ is the total magnetic flux when the coil is in its original state. Similarly, $t_{after}$ is the moment after the coil is fully stretched, and $t_{before}$ is the moment the stretching begins.

The total magnetic flux can be calculated as follows:

$\Phi=\oint_{S} \vec{B}\cdot\vec{S}ds$  **(S4)**

$\Phi_{before}=\sum_{i=1}^{225} \Phi_{i}$  **(S5)**

$\Phi_{after}=\sum_{j=1}^{225} \Phi_{j}$  **(S6)**

Where $\vec{B}$ and $\vec{S}$ represent the magnetic flux density vector and the area vector of the helix, respectively. The values of $\Phi_{after}$ and $\Phi_{before}$ can be calculated using Comsol Multiphysics software. the coil has 225 turns.

**References**

[1] Y. Zhou, X. Zhao, J. Xu, Y. Fang, G. Chen, Y. Song, S. Li, J. Chen, Giant magnetoelastic effect in soft systems for bioelectronics, Nat. Mater. 2021, 20, 1670.

[2] J. Xu, T. Tat, X. Zhao, Y. Zhou, D. Ngo, X. Xiao, J. Chen, A programmable magnetoelastic sensor array for self-powered human-machine interface, Appl. Phys. Rev. 2022, 9, 031404.

[3] X. Zhao, Y. Zhou, J. Xu, G. Chen, Y. Fang, T. Tat, X. Xiao, Y. Song, S. Li, J. Chen, Soft fibers with magnetoelasticity for wearable electronics, Nat. Commun. 2021, 12, 6755.

[4] G. Chen, X. Zhao, S. Andalib, J. Xu, Y. Zhou, T. Tat, K. Lin, J. Chen, Discovering giant magnetoelasticity in soft matter for electronic textiles, Matter 2021, 4, 3725.

[5] X. Zhao, A. Nashalian, I. W. Ock, S. Popoli, J. Xu, J. Yin, T. Tat, A. Libanori, G. Chen, Y. Zhou, J. Chen, A Soft Magnetoelastic Generator for Wind‐Energy Harvesting, Adv. Mater. 2022, 34, 2204238.

[6] J. Xu, T. Tat, X. Zhao, X. Xiao, Y. Zhou, J. Yin, K. Chen, J. Chen, Spherical Magnetoelastic Generator for Multidirectional Vibration Energy Harvesting, ACS Nano 2023, 17, 3865.

[7] Z. Ma, J. Ai, Y. Shi, K. Wang, B. Su, A Superhydrophobic Droplet‐Based Magnetoelectric Hybrid System to Generate Electricity and Collect Water Simultaneously, Adv. Mater. 2020, 32, 2006839.

[8] X. Zhang, J. Ai, Y. Yue, Y. Shi, R. Zou, B. Su, Anti-stress ball energy harvester, Nano Energy 2021, 90, 106493.

[9] H. Song, Y. Jang, J. P. Lee, J. K. Choe, M. Yun, Y.-K. Baek, J. Kim, Highly Compressible 3D-Printed Soft Magnetoelastic Sensors for Human-Machine Interfaces, ACS Appl. Mater. Interfaces 2023, 15, 59776.

[10] H. Wu, R. Luo, Z. Li, Y. Tian, J. Yuan, B. Su, K. Zhou, C. Yan, Y. Shi, Additively Manufactured Flexible Liquid Metal-Coated Self-Powered Magnetoelectric Sensors with High Design Freedom, Adv. Mater. 2023, 36, 2307546.

[11] I. W. Ock, X. Zhao, T. Tat, J. Xu, J. Chen, Harvesting Hydropower via a Magnetoelastic Generator for Sustainable Water Splitting, ACS Nano 2022, 16, 16816.

[12] W. He, M. Sohn, R. Ma, D. J. Kang, Flexible single-electrode triboelectric nanogenerators with MXene/PDMS composite film for biomechanical motion sensors, Nano Energy 2020, 78, 105383.

[13] L. Gu, J. Liu, N. Cui, Q. Xu, T. Du, L. Zhang, Z. Wang, C. Long, Y. Qin, Enhancing the current density of a piezoelectric nanogenerator using a three-dimensional intercalation electrode, Nat. Commun. 2020, 11, 1030.

[14] X. Wang, Q. Liu, X. Hu, M. You, Q. Zhang, K. Hu, Q. Zhang, Y. Xiang, Highly stretchable lactate-based piezoelectric elastomer with high current density and fast self-healing behaviors, Nano Energy 2022, 97, 107176.

[15] Y. Yao, X. Lu, C. Fu, Y. Zhang, J. Fang, J. Qin, Q. C. He, T. Yang, Patterned Coating of Ionic Diode Arrays Toward Flexible Moist‐Electric Generators to Power Wireless Sensor Nodes, Adv. Funct. Mater. 2023, 34, 2311465.

[16] H. Zhang, N. He, B. Wang, B. Ding, B. Jiang, D. Tang, L. Li, High‐Performance, Highly Stretchable, Flexible Moist‐Electric Generators via Molecular Engineering of Hydrogels, Adv. Mater. 2023, 35, 2300398.

[17] S. Liu, X. Liu, G. Zhou, F. Qin, M. Jing, L. Li, W. Song, Z. Sun, A high-efficiency bioinspired photoelectric-electromechanical integrated nanogenerator, Nat. Commun. 2020, 11, 6158.

[18] X. Yu, Y. Hou, Z. Yang, X. Gao, M. Zheng, M. Zhu, Boosting output current density of piezoceramic energy harvesters using three-dimensional embedded electrodes, Nano Energy 2022, 101, 107598.

[19] X. He, J. Zheng, T. Yang, D. Ou, C. R. Bowen, S. Shi, Q. Chen, H. Fu, S. Huang, Y. Ye, X. Huang, W. Liu, W. Yang, High-performance piezoelectric nanogenerators based on Cs_2_Ag_0.3_Na_0.7_InCl_6_ double perovskites with high polarity induced by Zr/Te codoping, Nano Energy 2023, 115, 108741.

[20] J. Bai, Y. Huang, H. Wang, T. Guang, Q. Liao, H. Cheng, S. Deng, Q. Li, Z. Shuai, L. Qu, Sunlight‐Coordinated High‐Performance Moisture Power in Natural Conditions, Adv. Mater. 2022, 34, 2103897.

[21] M. A. Johar, A. Waseem, M. A. Hassan, I. V. Bagal, A. Abdullah, J. S. Ha, S. W. Ryu, Highly Durable Piezoelectric Nanogenerator by Heteroepitaxy of GaN Nanowires on Cu Foil for Enhanced Output Using Ambient Actuation Sources, Adv. Energy Mater. 2020, 10, 2002608.

[22] J. Kim, H. Cho, M. Han, Y. Jung, S. S. Kwak, H. J. Yoon, B. Park, H. Kim, H. Kim, J. Park, S. W. Kim, Ultrahigh Power Output from Triboelectric Nanogenerator Based on Serrated Electrode via Spark Discharge, Adv. Energy Mater. 2020, 10, 2002312.

[23] L. Wang, W. A. Daoud, Highly Flexible and Transparent Polyionic‐Skin Triboelectric Nanogenerator for Biomechanical Motion Harvesting, Adv. Energy Mater. 2018, 9, 1803183.

[24] P. Slobodian, R. Olejnik, J. Matyas, P. Riha, B. Hausnerova, A coupled piezo-triboelectric nanogenerator based on the electrification of biaxially oriented polyethylene terephthalate food packaging films, Nano Energy 2023, 118, 108986.

[25] C. Yoon, S. Ippili, A. M. Thomas, B. Buyantogtokh, S. Hong, V. Jella, V.-D. Tran, S.-G. Yoon, Harnessing Flexoelectric and Piezoelectric Effects for Self-Charging Power Systems, ACS Energy Lett. 2023, 8, 4634.
